# Structural transformations in austenitic stainless steel induced by deuterium implantation: irradiation at 100 K

**DOI:** 10.1186/s11671-015-0852-0

**Published:** 2015-03-28

**Authors:** Oleksandr Morozov, Volodymyr Zhurba, Ivan Neklyudov, Oleksandr Mats, Aleksandr Rud, Nikolay Chernyak, Viktoria Progolaieva

**Affiliations:** National Science Center “Kharkiv Institute of Physics and Technology”, 1, Akademichna Street, 61108 Kharkiv, Ukraine; Institute for Metal Physics of NASU, 36 Academician Vernadsky Boulevard, 03142 Kharkiv, Ukraine

**Keywords:** Structural transformations, Austenitic stainless steel, Deuterium, Ion implantation, TDS, X-ray, TEM

## Abstract

Deuterium thermal desorption spectra were investigated on the samples of austenitic stainless steel 18Cr10NiTi preimplanted at 100 K with deuterium ions in the dose range from 3 **×** 10^15^ to 5 **×** 10^18^ D/cm^2^. The kinetics of structural transformation development in the implantation steel layer was traced from deuterium thermodesorption spectra as a function of implanted deuterium concentration. At saturation of austenitic stainless steel 18Cr10NiTi with deuterium by means of ion implantation, structural-phase changes take place, depending on the dose of implanted deuterium. The maximum attainable concentration of deuterium in steel is *C* = 1 (at.D/at.met. = 1/1). The increase in the implanted dose of deuterium is accompanied by the increase in the retained deuterium content, and as soon as the deuterium concentration attains *C* ≈ 0.5 the process of shear martensitic structural transformation in steel takes place. It includes the formation of bands, body-centered cubic (bcc) crystal structure, and the ferromagnetic phase. Upon reaching the deuterium concentration *C* > 0.5, the presence of these molecules causes shear martensitic structural transformations in the steel, which include the formation of characteristic bands, bcc crystal structure, and the ferromagnetic phase. At *C* ≥ 0.5, two hydride phases are formed in the steel, the decay temperatures of which are 240 and 275 K. The hydride phases are formed in the bcc structure resulting from the martensitic structural transformation in steel.

## Background

The investigation into regularities of hydrogen interaction with metals and alloys over a wide range of temperatures and pressures still remains a currently central problem in material physics from both the scientific and practical standpoints [1-9]. The hydrogen accumulation in structural and functional materials is an extremely hazardous phenomenon, which leads to hydrogen degradation of materials and to possible unforeseen equipment failures [10-14]. Degradation of materials increases due to hydrogen interaction with the whole range of crystal structure imperfections of solids such as interstitial and substitutional impurities, vacancies and their complexes, dislocations and their pileups, subgrain and grain boundaries, and phase components [11,15,16].

Vehoff [17] reviewed the interaction of hydrogen with defects in metals. He states that hydrogen concentrations far in excess of the lattice concentration cannot be produced by elastic interactions alone: the amount of hydrogen in a so-called Cottrell atmosphere around a dislocation is small. In contrast, chemical interaction or trapping at preferred lattice sites such as grain boundaries, particle-matrix interfaces, or dislocation cores can lead to a rise in local hydrogen concentration that can span many orders of magnitude. The respective concentration rise is highly dependent on the local chemical and/or structural arrangement [18-23].

Temperature, mechanical, radiation, and implantation effects give rise to additional permanent structural changes in metals, alloys, and steels. These are induced phase transformations, an increase in the density of dislocations and vacancies, pair production. All these factors enhance the hydrogen accumulation and lead to the loss of plasticity, and later on, to the failure [11,12,24-29].

Hydrogenation of the face-centered cubic (fcc) iron-based alloys, which constitute an array of austenitic stainless steels, can cause phase transformations: fcc (*γ*) → bcc (α*) and fcc (*γ*) → hcp (*ε*) [12,30-35]. The hydrogen-induced phases are sometimes considered as pseudohydrides. It is generally assumed that the role of hydrogen consists in creation of a particular stress state that triggers the phase transformation. In contrast, Vakhney and co-workers [36] showed that the phase transformation fcc (*γ*) → hcp (*ε*) was also due to a change in the electronic structure of the alloy after hydrogenation. At the same time, several studies have shown that martensite formation is responsible for hydrogen-enhanced crack growth, which results in quasi-cleavage fracture of specimens tested in hydrogen [37,38]. Gey and co-workers [39] found that the amount of strain-induced *ε*- and α*- martensite in AISI 304 steel (Pittsburgh, PA, USA) was strongly dependent on the local mutual orientations of neighboring grains, i.e., texture of the *γ* steel.

Stainless steel is one of the most useful classes of engineering materials. For example, austenitic steel is used for manufacturing vessel internals of fission-type reactors. A wide use of austenitic stainless steels as structural fission reactor elements calls for a detailed knowledge of their behavior under conditions of radiation influence, accumulation of gas impurities (hydrogen isotopes, above all) [12-14,19,26-29,40,41].

Note that the hydrogen effect on the martensitic phase transformations in austenitic stainless steels was observed only as a result of hydrogenation by the electrochemical method or from the gaseous phase. Furthermore, the hydrogen saturation of steel was performed both during and after different structure changing effects such as torsion, plastic deformation, dilatometry, and extrusion.

As to our knowledge, there are no literature communications about the observation of martensitic phase transformations in austenitic stainless steels resulting from implantation of hydrogen isotope ions, and about ultimate concentrations of hydrogen isotope retention in steels. Apparently, this is due to steel irradiation conditions, which impede the accumulation of high concentrations of hydrogen isotopes in austenitic stainless steels (irradiation temperature, ambient pressure of hydrogen, etc.).

One of the most informative methods of investigating hydrogen behavior in metals is the thermal desorption spectrometry (TDS). The TDS technique enables one to determine temperature ranges of implanted hydrogen retention and release, to find thermoactivation parameters, and to establish quantitative characteristics of hydrogen emission-re-emission. It also shows fair correlation between the thermoactivated hydrogen release spectra and the metal-hydrogen phase diagrams [42-44].

The present paper reports the results from studies on implanted deuterium-induced structural transformations in austenitic stainless steel 18Cr10NiTi as functions of implanted deuterium doses at a temperature of 100 K. This temperature provides a low diffusion mobility of deuterium, and hence, a high deuterium concentration in the implantation layer. The structural transformation kinetics has been traced from the deuterium thermodesorption spectra versus implanted deuterium concentrations. The important methodical advantage of the present study lies in the fact that the implantation of the assigned dose of deuterium and the measurements of temperature ranges of its desorption were performed in one and the same setup (*in situ*).

## Methods

The TDS technique has been used to investigate the kinetics of spectrum development for deuterium desorption from austenitic stainless steel 18Cr10NiTi versus the implanted deuterium dose. In our experiments, to reduce the impact of background hydrogen being present in the samples and in the target chamber, we have used the hydrogen isotope deuterium. The sample irradiation and the thermodesorption spectral measurements were carried out using the experimental facility ‘SKIF’ described in detail in ref. [45].

The samples were pre-implanted with 12 keV deuterium ions (${\mathrm{D}}_2^{+}$, 24 keV) of current density 5 μA/cm^2^ in the dose range from 3 **×** 10^15^ to 5 **×** 10^18^ D/cm^2^ at the sample temperature *T*_irr._ = 100 K. The low temperature was chosen to restrict the diffusion mobility of deuterium in the samples and to investigate its behavior in a wide range of concentrations produced in the implantation layer.

The samples were homogenized at 1,350 K for 30 min in a vacuum of 5 × 10^−5^ Pa. The austenitic stainless steel 18Cr10NiTi samples (Table 1), measuring 10 **×** 5 **×** 0.3 mm^3^, were fastened to the same-steel heaters, measuring 40 **×** 5 **×** 0.3 mm^3^. The radiation area was *S* = 0.3 cm^2^. Prior to irradiation, the samples underwent short-time annealing (1 min.) at 1,300 K in order to degas them and to remove impurities from their surface. After irradiation, the samples implanted to the pre-assigned dose were heated in the same measurement chamber at a rate of 3.5 K/s to a temperature of 1,700 K, with simultaneous registration of the ${\mathrm{D}}_2^{+}$ ion desorption spectrum (4 amu). The heating of samples started immediately after the ion beam was switched off. The temperature was measured by the tungsten-rhenium thermocouple WRe 5/20 fixed to the sample. The total amount of deuterium released from the sample was determined from the area lying under the gas release curve.

**Table 1:** **Chemical composition of austenitic stainless steel 18Cr10NiTi (weight percent)**

	**С**	**Cr**	**Ni**	**Ti**	**Mn**	**Si**	**P**	**S**	**Fe**
18Cr10NiTi	Approximately 0.1	17.1 to 18.5	9.6 to 10.2	0.45 to 0.56	1.0 to 1.2	0.4 to 0.43	0.03	0.01	Rest

The sample structure was examined by means of transmission electron microscopy at a voltage of 100 kV. The steel samples were thinned down to a thickness of 50 to 60 μm through chemical etch polishing, following which deuterium ions were implanted to the given dose. Thereafter, disks, 3 mm in diameter, were cut out from the obtained foil (the diameter was specified by the sample holder for the microscope), and a final thinning was done in the same solution.

The X-ray diffraction analysis of the samples was performed with the diffractometer HZG-4 (Seifert, Berlin, Germany) in the CuKα-radiation (β-filter).

The magnetic characteristics of 18Cr10NiTi steel and iron were measured with a bar-and-yoke permeameter. In this case, the sample in the form of a plate was placed in the axial (perpendicular to its plane) field.

## Results and discussion

### Kinetics of deuterium thermal desorption spectrum development in relation to the implanted deuterium dose

**Figure 1:**
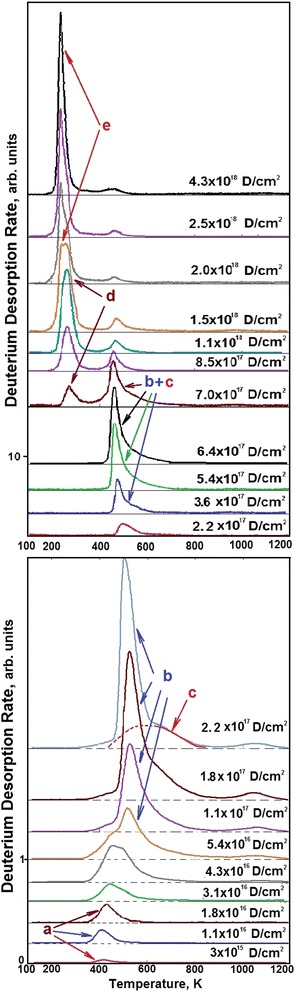
**Deuterium thermodesorption spectra from 18Cr10NiTi steel samples implanted with different doses of deuterium ions.**

**Figure 2:**
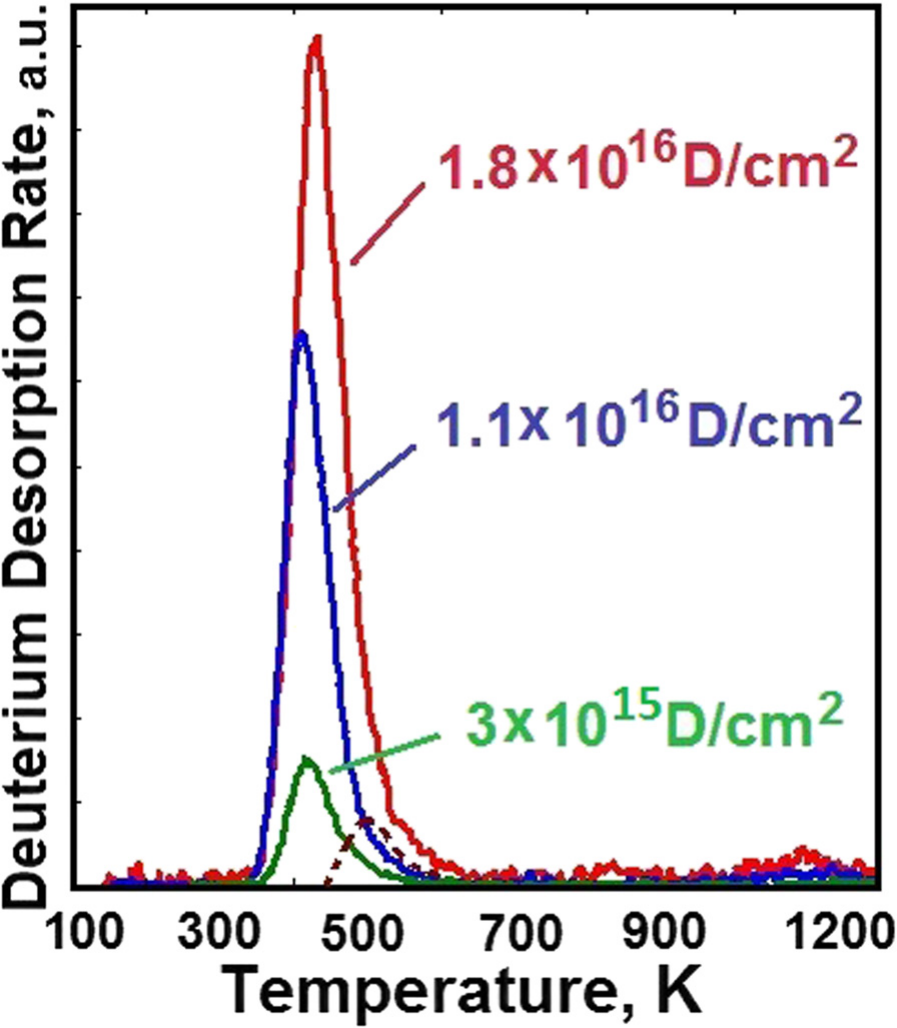
**Deuterium thermodesorption spectra from 18Cr10NiTi steel samples implanted to doses: 3 × 10** ^**15**^ **D/cm** ^**2**^ **; 1.1 × 10** ^**16**^ **D/cm** ^**2**^ **; 1.8 × 10** ^**16**^ **D/cm** ^**2**^ **.**

The most characteristic deuterium thermodesorption spectra for different implanted deuterium doses are shown in Figure 1. It can be seen that at low implantation doses (see Figure 1), the thermodesorption spectrum of ion-implanted deuterium represents a single peak with the maximum at *T*_m_ = 405 K, which we call the peak *a*. The presence of a single peak in the deuterium thermodesorption spectrum at low D concentrations suggests the conclusion that it characterizes the formation of the phase state of deuterium solid solution in the steel (see Figure 2). The appearance of the second peak in the thermodesorption spectrum points to the completion of the phase state formation of deuterium solid solution in steel. The deuterium concentration present in the implantation layer corresponds to ~2 at.% D for a dose of 1.8 **×** 10^16^ D/cm^2^.

At low deuterium concentrations, the second, higher-temperature peak (peak *b*, maximum temperature *T*_m_ = 500 K) is practically coincident with the low-temperature peak. An increase in the implanted deuterium dose is accompanied by enhancement in the peak intensity, and by the appearance of a moderate-intensity deuterium desorption region (extended in the temperature scale) at temperatures between 500 and 800 K (peak *c*). The presence of this region indicates that apart from the fractions with discrete values of thermodesorption activation energy, there is a gas flow, the activation energy of which smoothly varies in wide temperature ranges. We identify this temperature scale-extended region of deuterium desorption as an amorphous phase state of the solid, the presence of which is necessary for structural transformations.

The excess of the implanted deuterium dose 1.8 **×** 10^17^ D/cm^2^ is accompanied by a gradual decrease in the *b* peak maximum temperature, which becomes equal to 430 K at a dose of 6.4 **×** 10^17^ D/cm^2^. At the same time, as the implantation dose increases, an essential change takes place in the geometric shape of this thermodesorption spectral peak. It takes on a characteristic form with a sharp, nearly vertical, front, and a slowly falling-off high-temperature part.

The observed decrease in the temperature of the peak *b* maximum can be mainly due to both the stress buildup owing to a growing concentration of implanted deuterium, and the presence of the amorphous component in the steel structure (peak *c*). This temperature region appears practically simultaneously with the second peak in the deuterium thermodesorption spectrum; it has the maximum intensity (amount of desorbed deuterium in the peak *c*) at the dose 6.4 **×** 10^17^ D/cm^2^ and shows a decrease in the intensity with a further increase in the implanted deuterium dose.

At doses higher than 6.5 **×** 10^17^ D/cm^2^, the deuterium thermodesorption spectrum qualitatively changes, this being manifested in the appearance of a low-temperature region of deuterium desorption, which testifies to the emergence of a new phase state; that may be considered as a hydride formation. The conclusion about hydride formation was made on the basis of our data obtained when studying the deuterium thermodesorption spectra from Pd [42] and Ti [43]. It was indicated there that in the deuterium thermodesorption spectrum, the hydride formation showed itself as the occurrence of lower-temperature peaks.

From the given TDS spectra, it can be seen that at the irradiation dose 7 **×** 10^17^ D/cm^2^, apart from the previously observed peaks *b* and *c*, there is only one, strongly blurred peak centered at 275 K, which is due to the hydride formation (peak *d*). With an increasing dose, this peak grows in its amplitude, and the spectrum exhibits one more peak with the maximum at 240 K (peak *e*). At 1.5 **×** 10^18^ D/cm^2^, the amplitudes of these low-temperature peaks become practically equal (see Figure 1). A further increase in the implanted deuterium dose leads to a rapid increase in the intensity of the lowest-temperature peak of the deuterium thermodesorption spectrum, with the result that the peak becomes prevailing. In this case, the intensities of the peaks *b* and *d* decrease. The presence of clearly defined peaks *d* and *e* in the deuterium thermodesorption spectra suggests an important conclusion that in the steel-deuterium system, there are two hydride phases degrading at temperatures of 240 and 275 K. It is also obvious from Figure 1 that the lowest-temperature hydride phase (peak *e*) is formed due to both the newly implanted deuterium and the earlier formed higher-temperature phases (peaks *d*, *b*, and *c*).

Note that the formation of low-temperature regions of deuterium desorption is accompanied by the appearance of the deuterium desorption region, extended in the temperature scale in the range between 270 and 430 K. This provides additional supporting evidence that the amorphous phase state of the solid is necessary for the realization of structural changes.

### Amount of deuterium in different phase states of the deuterium-steel system

**Figure 3:**
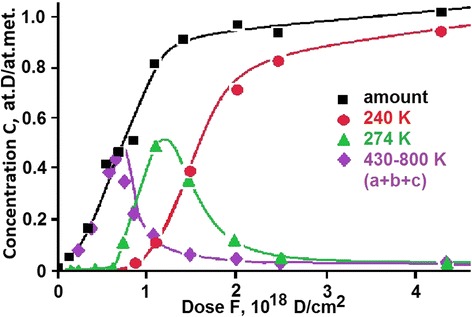
**Deuterium content in each individual peak of the spectrum and the integral quantity of desorbed deuterium as functions of radiation dose for austenitic steel.**

Using the deuterium thermodesorption spectra from the 18Cr10NiTi steel samples exposed to different doses, we have plotted the total amount of deuterium *C*(*F*) desorbed from the sample as a function of the radiation dose *F*. This function is presented in Figure 3. The figure also shows the dose dependences of the amount of deuterium desorbed from three most intense peaks observed in the TDS spectra.

The linear dependence of the amount of implanted deuterium on the irradiation dose value persists until the dose *F* = 1.3 **×** 10^18^ D/cm^2^ is reached. Then we observe an abrupt departure from the linearity and the tendency for attaining saturation at a dose of 1.5 **×** 10^18^ D/cm^2^, though the true saturation is not attained. In this case, the excessive deuterium is mostly desorbed from steel, and only a small part of deuterium reaches the steel deuteride-steel interface, thereby providing the deuteride penetration deep into the sample, and also, the increase in the amount of trapped deuterium.

The concentrations of implanted deuterium were estimated with due regard for both the amount of metal atoms in the implanted layer and the amount of implanted deuterium. In this case, it was assumed that implanted deuterium had low diffusion mobility in the steel cooled down to 100 K, and practically all deuterium was in the implantation layer. The estimation has shown that saturation of 18Cr10NiTi steel with deuterium is attained at deuterium concentration *C* (at.D/at.met.) ≈ 1.0. When plotting the *C*(*F*) curve, it was taken into account that the steel surface reflection coefficient for the D^+^ ions of energy 12 keV per deuteron was about 10% [46].

At the initial stage of implantation dose increase, we observe the formation of the phase state of deuterium solid solution in steel (peak *a* with the maximum temperature 405 K at *C* = 0.02 at.D/at.met. = 2 at.% deuterium), and also, the emergence of two regions of deuterium desorption in the temperature range between 430 and 800 K (see Figure 1, peaks *b* and *c*), the total concentration of which reaches *C* = 0.5 at.D/at.met.

The excess of deuterium concentration in steel *C* ≥ 0.5 at.D/at.met. gives rise to the low-temperature region of deuterium desorption. First, the hydride phase is formed, which degrades at 275 K (peak *d* in Figure 1), and then, with some delay in the dose, the second hydride phase is formed, with the degradation temperature of 240 K (peak *e*). The increase in the amount of deuterium in the hydride phase that degrades at 275 K (peak *d* in Figure 1) continues until the concentration *C* = 0.5 at.D/at.met. is attained. At that, the intensity of the earlier formed higher-temperature phases (peaks *b* and *c*) decreases.

The formation of the lowest-temperature hydride phase that decays at 240 K (peak *e*) occurs due to both the newly implanted deuterium and the earlier produced higher-temperature phases (peaks *d*, *b*, and *c*). The highest deuterium concentration in the low-temperature phase of steel is attained at *C* = 0.9, this approaching the stoichiometry at.D/at.met. = 1/1.

### On the nature of deuterium retention in the 18Cr10NiTi steel

From the analysis of the development kinetics of the deuterium thermodesorption spectrum versus the dose of implanted deuterium, it follows that after formation of the phase state of deuterium solid solution in the 18Cr10NiTi steel, a higher-temperature phase state is formed, which is not typical of hydride-forming metals. Note that the hydride-forming metals are characterized by the formation of hydrides having a lower decay temperature of the phase state of deuterium solid solution in the metal [42,43]. This manifests itself in the thermodesorption spectra of deuterium by the emergence of lower-temperature peaks. This result points to different nature of deuterium retention in the austenitic stainless steel 18Cr10NiTi and in the hydride-forming metals. The data suggest an important conclusion that the austenitic steel, having the fcc structure, forms no hydrides.

It is then quite logical to assume that the phase state with the maximum temperature 500 K (peak *b*, Figure 1) represents the kinetics of austenitic stainless steel lattice occupation without formation of a chemical compound (hydride). From the data on the amount of deuterium desorbed (see Figure 2) from the steel in this phase state, one can obtain the deuterium concentration value, which corresponds to *C* = 0.5 at.D/at.met. = 1/2.

**Figure 4:**
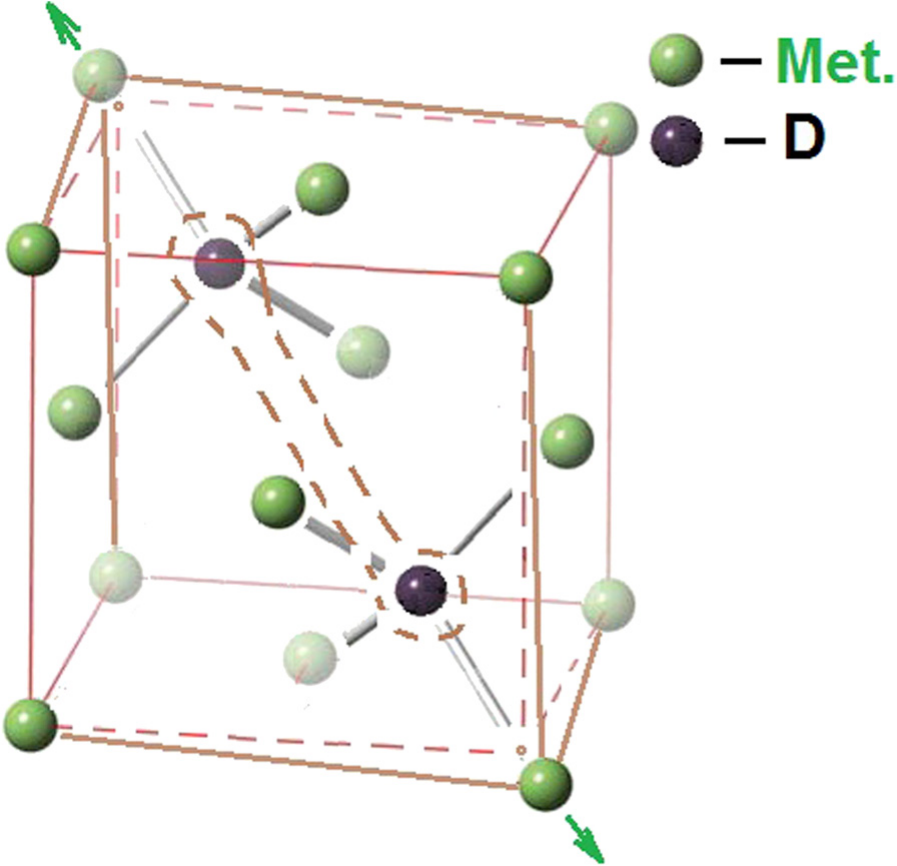
**Crystalline structure of austenitic stainless steel with deuterium at** ***C*** **= 0.5 at.D/at.met.**

Based on these data, the following scheme of deuterium atoms arrangement in the fcc lattice of steel can be offered: two deuterium atoms are arranged diagonally in two out of eight tetrahedral sites, producing a strong distortion along the (111) axis of the fcc lattice (see Figure 4). With occupation of practically all elementary cells of the implantation steel layer with deuterium, and with attainment of the concentration *C* ≥ 0.5 at.D/at.met. = 1/2, a shear martensitic structural transformation and the relaxation of implanted deuterium-induced stressed state take place.

**Figure 5:**
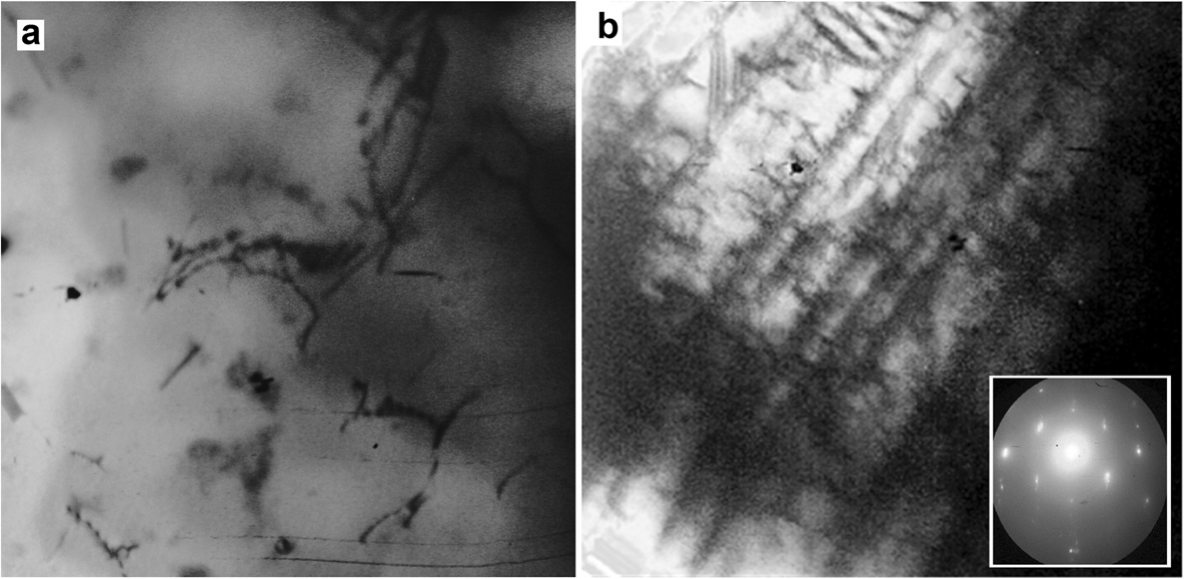
**Structure of 18Cr10NiTi steel: (a) initial and (b) after deuterium ion irradiation to a dose of 8.2 × 10** ^**17**^ **D/cm** ^**2**^ **.** ×90,000 magnification. The inset in **(b)** shows the electron diffraction pattern.

The electron microscope studies of the samples (see Figure 5) have confirmed the realization of the process of shear martensitic structural transformation on attainment of the concentration *C* ≥ 0.5 at.D**/**at.met.

The electron-microscopical images of the initial samples exhibit the peculiar to the austenitic stainless steel large-size crystallites, and the randomly distributed inclusions scattered all over the sample (Figure 5a). As it is obvious from Figure 5b, in the surface layer of the steel irradiated with deuterium ions, there are austenites, the needles of *α*-martensite and *ε***-**interlayer, the presence of which testifies to the actual shear polymorphous transformations realized through the intermediate *ε*-hcp phase: *γ* **→** *ε* **→** *α.*

**Figure 6:**
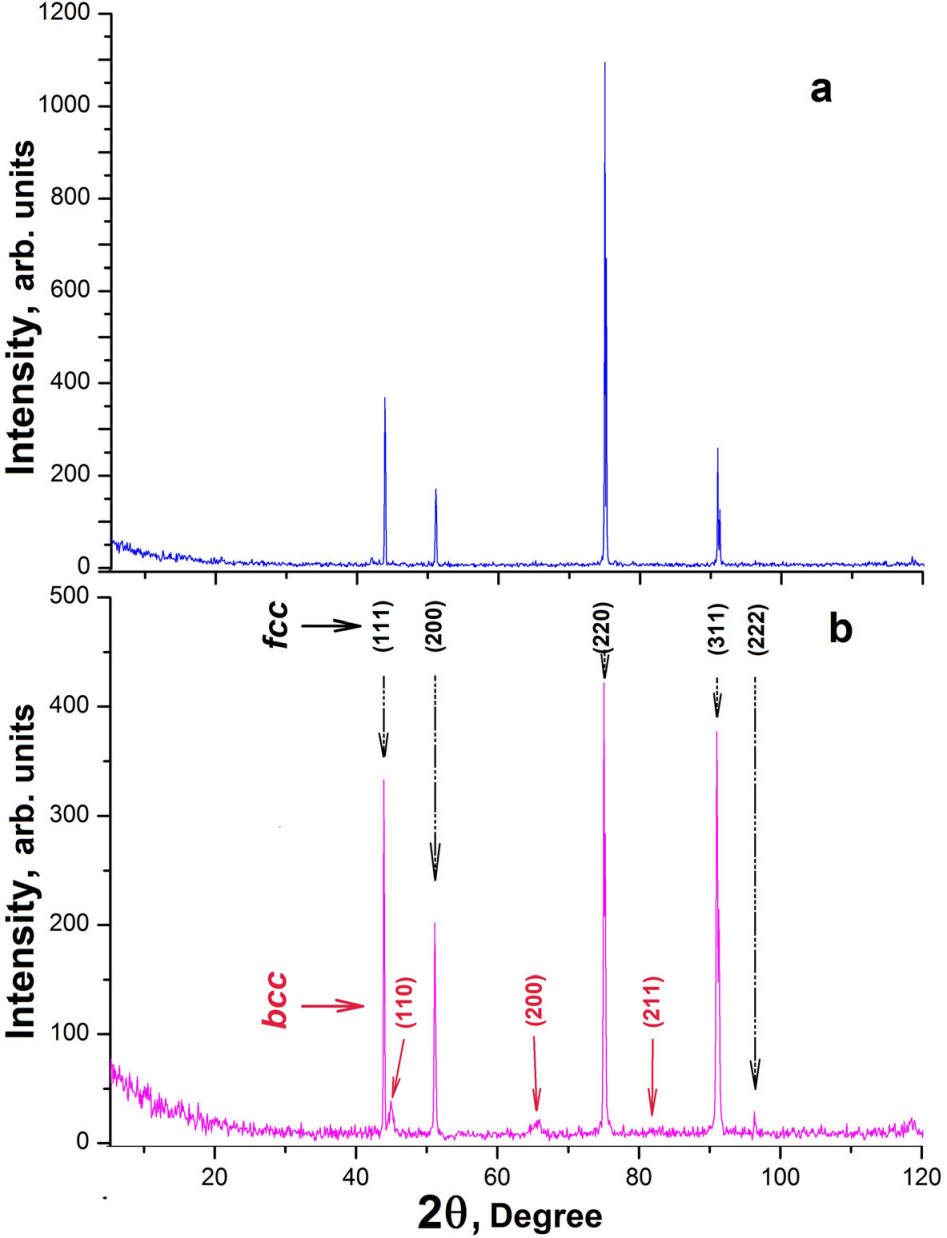
**Diffractograms of 18Cr10NiTi steel: (а) initial and (b) irradiated with deuterium ions to 8.2 × 10** ^**17**^ **D/cm** ^**2**^ **.**

X-ray diffraction studies of similar samples (see Figure 6) also provide evidence for the realization of the process of the shear martensitic structural transformation on attainment of the deuterium concentration *C* ≥ 0.5 at.D/at.met., namely, the appearance of the body-centered cubic (bcc) structure has been found against the background of intense lines of the fcc structure of the 18Cr10NiTi steel. A low bcc phase intensity is due to the fact that the approximately 150 nm thick implantation layer, where phase changes have taken place, is substantially smaller in thickness than the sample under study.

**Figure 7:**
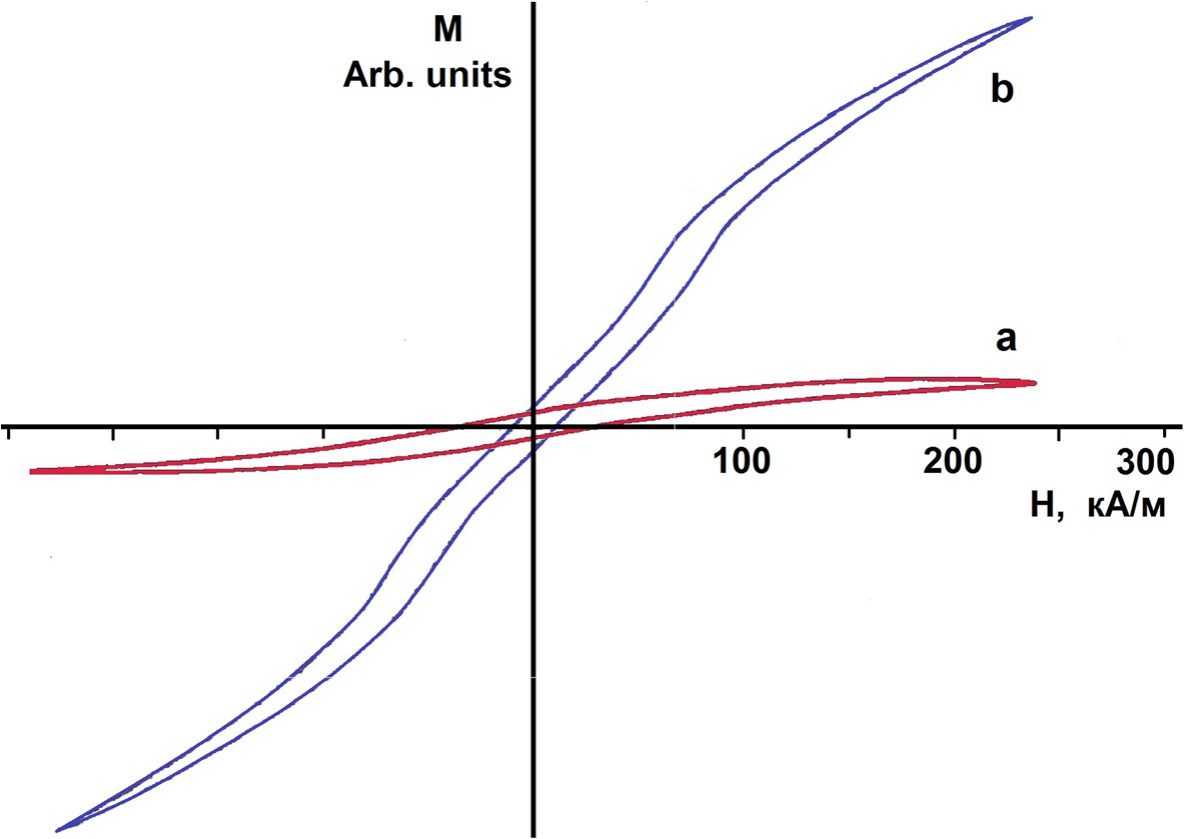
**Magnetization curves of the samples,** ***М*** **(** ***Н*** **):** **(а) 18Cr10NiTi steel implanted with deuterium ions to 1 × 10** ^**18**^ **D/cm** ^**2**^ **at 100 K;** **(b) Armco iron sample.**

An additional verification of the realization of the process of shear martensitic structural transformation on attainment of the deuterium concentration C ≥ 0.5 at.D/at.met. is provided by measurements of the magnetic characteristics of steel. For comparative tests, samples from the 18Cr10NiTi steel and Armco iron (AK Steel International, Stevenage, Great Britain) were prepared as plates nearly of the same size, the difference being no more than 1%. The measurements were carried out using the bar-and-yoke permeameter. In this case, the sample was arranged in the axial (perpendicular to its plane) field. Figure 7 depicts the magnetization curves (*M*(*H*)) of the samples under study. The initial 18Cr10NiTi steel samples, which underwent austenitizing annealing, show up no hysteresis dependence typical of ferromagnetic materials. The examination of steel samples implanted with deuterium ions to a dose of 1 **×** 10^18^ D/cm^2^ at a temperature 100 K has revealed the appearance of the hysteresis dependence typical of a ferromagnetic (see Figure 7, curve a). To estimate the ferromagnetic phase concentration resulting from deuterium implantation, the experiment was made to investigate the Armco iron magnetization (see Figure 7, curve b). The comparative analysis has led to the conclusion that the ferromagnetic phase formed in the 18Cr10NiTi steel during deuterium implantation is no more than 2% to 3% of the sample volume (with due regard for the experimental error). The low intensity of the registered ferromagnetic phase is due to the fact that the implantation layer, where structural-phase transformations of the fcc structure (with no ferromagnetic phase) into the bcc structure (having hysteresis dependence typical of ferromagnetics) took place, has the thickness considerably smaller than that of the sample under study.

Nonetheless, the estimates have shown that the ferromagnetic phase formed in the 18Cr10NiTi steel during deuterium implantation exceeds the implantation layer thickness by nearly an order of magnitude. Hence, the process of the shear martensitic structural transformation on attainment of the deuterium concentration *C* ≥ 0.5 at.D/at.met. is realized to the depth exceeding the implantation layer thickness.

Notice that the bcc structure formation in the 18Cr10NiTi steel occurs simultaneously with the appearance of low-temperature peaks. These data permit us to conclude that the hydrides in the austenitic stainless steel are produced only after the bcc structure inclusions are formed in the steel, and, as a consequence, it follows that the structure of the steel hydride sublattice represents the bcc structure.

**Figure 8:**
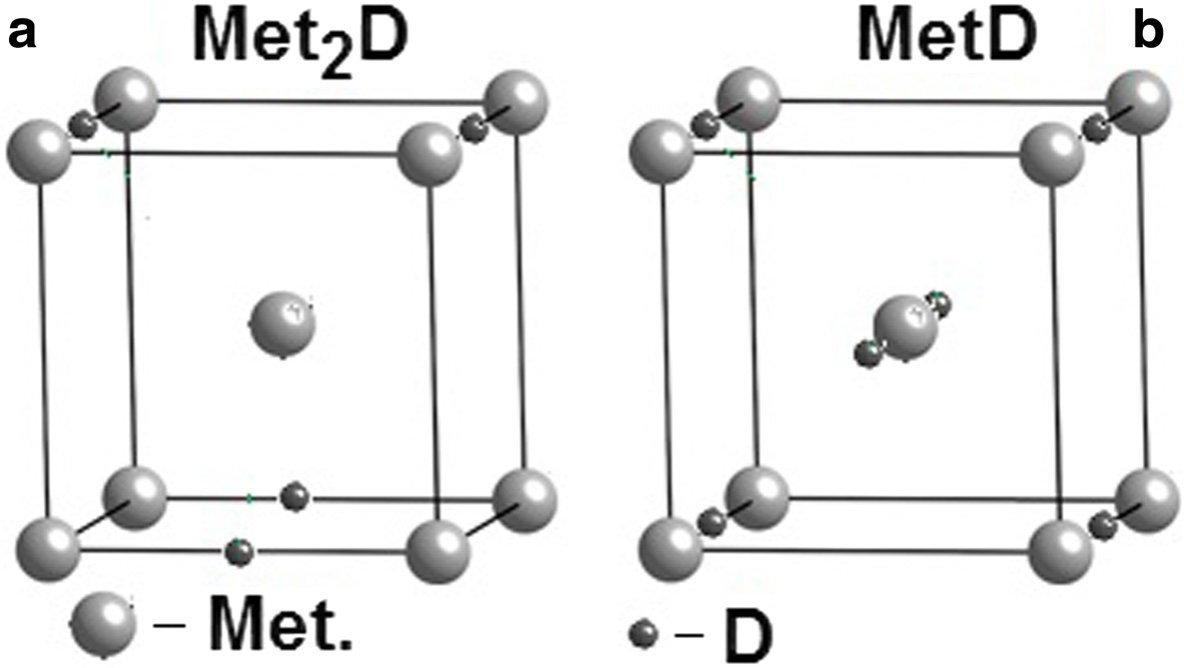
**Crystalline structure of austenitic stainless steel with deuterium concentrations (** **а)** ***С*** **= 0.5 at.D/at.met. and (** **b)** ***С*** **= 1 at.D/at.met.**

Based on the belief that deuterium atoms in the bcc crystal lattice of steel are in the charged state, are in octahedral sites, and at regular intervals, one can elaborate the arrangement of deuterium atoms in the hydride phase of steel with the deuterium concentration *C* = 1/2 at.D/at.met. = 0.5 (see Figure 8a, peak *d* in Figure 1). A further increase in the implanted deuterium concentration is accompanied by the formation of the lower-temperature hydride phase in the same crystal lattice, where the hydride structure with deuterium concentration *C* = 0.5 has already formed. This phase is formed due to both the newly implanted deuterium, and the earlier formed higher-temperature hydride phase (peak *d*). In this case, the local concentration of deuterium in the steel-lattice crystal structure reaches *C* = 1 at.D/at.met. (see Figure 8b, peak *e* in Figure 1). The decay of this lowest-temperature hydride phase in the deuterium thermodesorption spectrum is registered as a single peak. Note that in the Pd-D system, two stable hydride sublattices in palladium are also formed [42].

The important conclusion that follows from the present experimental data is that *the appearance of a low-temperature region of deuterium desorption from steel is connected with the α-phase formation*, i.e., the formation of the bcc structure typical of the low-temperature phase of iron, being the principal chemical component of the austenitic stainless steel.

The second important conclusion is that the hydride phase formation occurs not until the bcc structure has developed. Then, it follows that, firstly, in the fcc structure of the 18Cr10NiTi steel hydrides are not formed, and secondly, the metal sublattice of the hydride phases of 18Cr10NiTi steel has the bcc structure.

Notice that the formation of bcc structure inclusions in the austenitic stainless steel has been observed using the TEM technique, the X-ray diffraction method; the measurements of magnetic properties (see Figures 4, 5, and 6). These data provide an additional support for the fact that the peaks in the deuterium thermodesorption spectra are related with the phase transformations in metals.

The results of the studies have demonstrated that the amount of the retained deuterium is dependent on the structural-phase state of the steel.

At saturation of austenitic stainless steel 18Cr10NiTi with deuterium through ion implantation at a temperature of 100 K, there occur structural-phase changes, dependent on the implanted deuterium dose. At low implantation doses, the phase state of deuterium solid solution in the steel is formed, the deuterium concentration being 2 to 4 at.%. With an increase in the implanted deuterium dose, the concentration of the retained deuterium increases. The ultimate concentration of deuterium retention in the fcc crystal lattice of austenitic steel attains *C* = 0.5 at.D/met. Deuterium occupies interstitial fcc lattice sites of the austenitic steel.

A further increase in the implantation dose leads to the martensitic structural transformation in the steel with the result that the fcc crystal lattice of the steel changes into bcc lattice (this has been registered by the methods of TEM, X-ray diffraction, and by measurements of magnetic properties). The deuterium concentration increase in the bcc lattice of steel continues with an increase in the implantation dose up to *C* = 1 at.D/met. In this case, deuterium is found in the octahedral interstitial bcc lattice sites of the steel, occupying half of the available ones.

The TDS technique was used to determine the deuterium concentration in the phase states, and also to trace the process of structural transformations in the steel (see Figures 2 and 3).

## Conclusions

At saturation of the austenitic stainless steel with deuterium through ion implantation at a temperature of 100 K, structural-phase changes take place, depending on the implanted deuterium dose.

The highest attainable concentration of deuterium in steel has been determined to be *C* = 1 at.D/at.met.

The increase in the implanted dose of deuterium is accompanied by the increase in the retained deuterium content, and as soon as the deuterium concentration attains *C* ≈ 0.5, the process of shear martensitic structural transformation in steel takes place. It includes the formation of bands, bcc crystal structure, and the ferromagnetic phase.

At *C* ≥ 0.5, two hydride phases are formed in the steel, the decay temperatures of which are 240 and 275 K. The hydride phases develop in the steel bcc structure, which results from the martensitic structural transformation.

The present data have confirmed that the peaks observed in the deuterium thermodesorption spectra are related to the phase transformations in metals. The kinetics of structural transformations development in the implantation layer of steel has been traced from the spectra of deuterium thermal desorption as a function of implanted deuterium concentration.

## References

[1] Alefeld G, Völkl J (1978). Hydrogen in metals I.

[2] Alefeld G, Völkl J (1978). Hydrogen in metals II.

[3] Wipf H (1997). Hydroqen in metals III. Properties and applications.

[4] Gel’d PV, Ryabov RA, Kodes ES (1979). Hydrogen and metal structure imperfections (Russian).

[5] Ageyev VN, Bekman IN, Gol’tsov VA (1987). Hydrogen interaction with metals (in Russian).

[6] Lewis FA, Aladjem A (2000). Hydrogen in metal systems. II.

[7] Varin RA, Czujko T, Wronski ZS (2009). Nanomaterials for solid state hydrogen storage.

[8] Zuttel A, Borgschulte A, Schlapbach L (2008). Hydrogen as a future energy carrier.

[9] Broom DP (2011). Hydrogen storage materials. The characterization of their storage properties.

[10] Kolachev BA (1985). Hydrogen embrittlement of metals (in Russian).

[11] Gol’tsov VA (2001). Progress in hydrogen treatment of materials.

[12] Borchers C, Michler T, Pundt A (2008). Effect of hydrogen on the mechanical properties of stainless steels. Adv Eng Mater.

[13] Touge M, Miki T, Ikeya M (1983). Effects of X-ray irradiation on hydrogen-induced phase transformations in stainless steel. Metall Trans.

[14] Neklyudov IM, Ozhigov LS, Shilyayev BA, Laptev IN, Parkhomenko АА, Morozov AN, et al. Hydrogen in stainless steel in-vessel components of the WWER-1000 reactor (in Russian). Voprosy At Nauki I Tekhniki Series: Fizika radiats povr i radiats materialoved. 2003;83:47–50.

[15] Myers SM, Baskes MI, Birnbaum HK, Corbett JW, DeLeo GG, Estreicher SK (1992). Hydrogen interactions with defects in crystalline solids. Rev Modern Phys.

[16] Gavriljuk VG (2006). Austenite and martensite in nitrogen-, carbon- and hydrogen-containing iron alloys: similarities and difference. Mater Sci Eng A.

[17] Vehoff H, Wipf H (1997). Hydrogen related material problems. Hydrogen in metals III.

[18] Čížek J, Procházka I, Becvár F, Kužel R, Cieslar M, Brauer G (2004). Hydrogen-induced defects in bulk niobium. Phys Rev.

[19] Yoshida N, Ashizuka N, Fujiwara T, Kurita T, Muroga T (1988). Radiation damage and deuterium trapping in deuterium ion irradiated austenitic stainless steel. J Nucl Mater.

[20] Yoshida N, Kurita T, Fujiwara T, Muroga T (1989). Trapping of deuterium injected in austenitic stainless steel at elevated temperatures. J Nucl Mater.

[21] Čížek J, Procházka I, Brauer G, Anwand W, Mücklich A, Kirchheim R (2006). Defect studies of hydrogen-loaded thin Nb films. Appl Surf Sci.

[22] Gussev MN, Busby JT, Tan L, Garner FA (2014). Magnetic phase formation in irradiated austenitic alloys. J Nucl Mater.

[23] Shirbanda Z, Shishesaza MR, Ashrafib A (2011). Hydrogen degradation of steels and its related parameters, a review. Phase Transit.

[24] Bryk VV, Neklyudov IM. Regularities of dislocation structure evolution in self-organizing materials (in Russian). Voprosy At Nauki I Tekhniki Series: Fizika radiats povr i radiats materialoved. 2001;80:9–13.

[25] Rudenko AG, Shilyaev BA, Voyevodin VN, Ozhigov LS. Evolution of the radiation damage materials of the reactor WWER-1000 (in Russian). Voprosy At Nauki I Tekhniki Series: Fizika radiats povr i radiats materialoved. 2008;92:78–82.

[26] Neklyudov IM, Morozov AN, Zhurba VI, Kulish VG, Galitsky AG. Hydrogen isotope retention in 18Cr10NiТi steel implanted with helium ions (in Russian). Voprosy At Nauki I Tekhniki Series: Termoyadernyi sintez. 2008;2:41–6.

[27] Chernov IP, Martynenko YV, Cherdantsev YP. Mutual influence of hydrogen and helium in structural materials (in Russian). Voprosy At Nauki I Tekhniki Series: Termoyadernyi sintez. 2008;2:46–50.

[28] Neklyudov I, Morozov O, Kulish V, Azhazha V, Lavrinenko S, Zhurba V (2011). The effects of helium on temperature ranges of hydrogen isotopes retention in Hastelloy-N alloy. J Nucl Mater.

[29] Neklyudov IM, Morozov AN, Kulish VG, Zhurba VI, Galytsky AG, Piatenko EV (2009). The influence of interstitial impurities on temperature ranges of deuterium retention in austenitic stainless steel. J Nucl Mater.

[30] Benson RB, Dann RK, Roberts J (1968). Hydrogen embrittlement of stainless steel. Trans Metal Soc AIME.

[31] Teus SM, Shyvanyuk VN, Gavriljuk VG (2008). Hydrogen-induced *γ → ε* transformation and the role of *ε*-martensite in hydrogen embrittlement of austenitic steels. Mater Sci Eng.

[32] Kishi A, Takano N (2010). Effect of hydrogen cathodic charging on fatigue fracture of type 310S stainless steel. J Phys Conf Ser.

[33] Mine Y, Horita Z, Murakami Y (2009). Effect of hydrogen on martensite formation in austenitic stainless steels in high-pressure torsion. Acta Mater.

[34] Rozenak P, Eliezer D (1988). Nature of the *γ* and *γ** phases in ustenitic stainless steels cathodically charged with hydrogen. Metal Trans.

[35] Rozenak P, Bergman R (2006). X-ray phase analysis of martensitic transformations in austenitic stainless steels electrochemically charged with hydrogen. Mater Sci Eng.

[36] Vakhney AG, Yaresko AN, Antonov VN, Nemoshkalenko VV (2001). The effect of hydrogen on the electronic structure and cohesive properties of iron-based alloys doped by chromium and nickel. Int J Hydrogen Energy.

[37] Tsay LW, Liu YC, Young MC, Lin D-Y (2004). Fatigue crack growth of AISI 304 stainless steel welds in air and hydrogen. Mater Sci Eng.

[38] Tsay LW, Yu SC, Huang RT (2007). Effect of austenite instability on the hydrogen-enhanced crack growth of austenitic stainless steels. Corros Sci.

[39] Gey N, Petit B, Humbert M (2005). Electron backscattered diffraction study of ε/α′ martensitic variants induced by plastic deformation in 304 stainless steel. Metal Mater Trans.

[40] Wilson KL, Baskes MI (1978). Thermal desorption of deuterium implanted stainless steel. J Nucl Mater.

[41] Pontau AE, Baskes MI, Wilson KL, Haggmark LG, Bohdansky J, Scherzer BMU (1982). Deuterium retention in helium-damaged stainless steel: detrapping energy. J Nucl Mater.

[42] Rybalko VF, Morozov AN, Neklyudov IM, Kulish VG (2001). Observation of new phases in Pd-D systems. Phys Lett.

[43] Neklyudov IM, Morozov AN, Kulish VG. Temperature ranges of hydride phase stability of the TiD system (in Russian). Materialovedeniye. 2005;№11:45–56.

[44] Escobar DP, Depover T, Duprez L, Verbeken K, Verhaege M (2012). Combined thermal desorption spectroscopy, differential scanning calorimetry, scanning electron microscopy and X-ray diffraction study of hydrogen trapping in cold deformed TRIP steel. Acta Mater.

[45] Ruzhitsky VV, Gribanov YA, Rybalko VF, Khazan SM, Morozov AN, Martynov IS. The multipurpose experimental facility “SKIF” (in Russian). Voprosy At Nauki I Tekhniki Series: Fizika radiats povr i radiats materialoved. 1989;51:84–9.

[46] Möller W, Besenbacher F, Bottiger J (1982). Saturation and isotope mixing during low-temperature implantations of hydrogen into metals. Appl Phys A.

